# Self-Propagating Heat Synthetic Reactivity of Fine Aluminum Particles via Spontaneously Coated Nickel Layer

**DOI:** 10.1038/s41598-018-36760-y

**Published:** 2019-01-31

**Authors:** Dong Won Kim, Kyung Tae Kim, Gu Hyun Kwon, Kyung Song, Injoon Son

**Affiliations:** 1 Korea Institute of Materials Science, 797 Changwondaero, Seongsan-gu, Changwon, Gyeongnam 51508 Republic of Korea; 2 Kyungpook National University, 80 Daehakro, Buk-gu, Daegu 41566 Republic of Korea

## Abstract

Aluminum powders are known to provide outstanding volumetric exothermic enthalpy energy during thermal oxidation. However, the amount of energy released tends to be limited by the dense surface oxide (Al_2_O_3_) layer of the powder. Hence, a prerequisite for improving the reactivity of passivated Al particles is to remove the Al_2_O_3_ film from the surface. Considering that the self-propagating high-temperature synthesis (SHS) reaction of Ni and Al can generate additional exothermic heat in Al powder, Ni can be considered as a promising alternative to the surface oxide layer. Here, we report oxide-layer-free fine Al particles with a characteristic Ni/Al interface, where a Ni layer replaces the Al_2_O_3_ film. The microstructure of the synthesized powder consists of a 200-nm-thick Ni layer homogeneously coated on the Al surface, which has nanosized craters caused by the geometrical removal of Al_2_O_3_. Thermal analysis and *in-situ* heating transmission electron microscopy (TEM) results clearly show that active interdiffusion of atoms through the Ni/Al interface results in the formation of intermetallic compounds to provide additional exothermic energy, compared to the result for simply mixing Ni and Al powders. Hence, these findings provide new routes for the design and application of reactive metallic particles using the SHS reaction.

## Introduction

Aluminum exhibits excellent energy release characteristics in oxidation reactions compared to other metallic materials^1–6^. As its specific surface area is maximized, Al powder can find application as an energetic material in solid fuels, propellants, and brazing materials^4–9^. However, Al is known to form a spontaneously passivated oxide surface layer in a natural environment; the resulting Al_2_O_3_ layer (melting point: approximately 2350 K) hinders direct reaction between ambient oxygen and the Al, thus lowering the oxidation efficiency^6,8–11^. To overcome this limitation on the exothermic reactivity, many researchers have developed technologies for a pre-ignition reaction or surface modification with organic or inorganic materials. Several candidates such as fluorine-based polymers^9–13^, epoxy-based systems^2,14^, iron^15^, palladium^16^, and nickel^8,16–20^ have shown promising results in terms of efficient ignition and combustion. In particular, Ni materials have attracted significant interest for use as a coating material or composite film for Al, owing to the notable advantage of the Al–Ni system for inducing the self-propagating high-temperature synthesis (SHS) reaction of the two metals^3,8^. In addition, fundamental research has revealed that the heat energy in SHS originates from the formation of intermetallic compounds such as NiAl or NiAl_3_^21–26^.

On the other hand, the rate of thermal oxidation of Al particles is known to increase with decreasing particle size owing to the resulting high specific surface area. However, for nanometer-sized Al particles, the total amount of energy released is reduced because of the higher amount of Al oxide. That is, for 30-nm Al particles, the Al core makes up less than 50% of the total powder because the thickness of the surface oxide layer is generally approximately 4 nm^6,27^. Hence, considering the combination of rapid ignition and a large energy release, fine Al powders with particle sizes ranging from 1 to 10 μm are suitable for practical energetic applications, for example, in metal fuels.

Despite the expected advantages of Al powder in this size range, improvement of the exothermic reactivity by directly coating Ni materials onto fine Al particles has not been reported. The reason may be the experimental difficulty of preventing the re-oxidation of the Al particles during coating owing to the stepwise *ex-situ* process adopted. In particular, it is necessary to ensure direct contact between Ni and Al rather than induce the formation of Ni/Al_2_O_3_/Al interface to realize rapid ignition as well as active SHS. Furthermore, obtaining a geometrically large area at the interface facilitates effective atom diffusion during oxidation.

Here, we report the spontaneous formation of a 200-nm-thick Ni layer on the surface of Al powder containing nanosized craters that are created during the removal of the surface oxide. The Al powder used in this study has a particle size of approximately 5 μm, and the coated material consists of mainly Ni and 14 wt% P. The microstructure of the Ni-coated Al (Ni/Al) powder was analyzed with an emphasis on the Ni/Al interface to confirm the direct contact between, and the interfacial morphology of the two materials. The thermochemical behavior of the Ni/Al powder as a function of temperature was compared with those of alumina-passivated Al and Al powder simply mixed with 200-nm Ni powder.

## Results and Discussion

**Figure 1:**
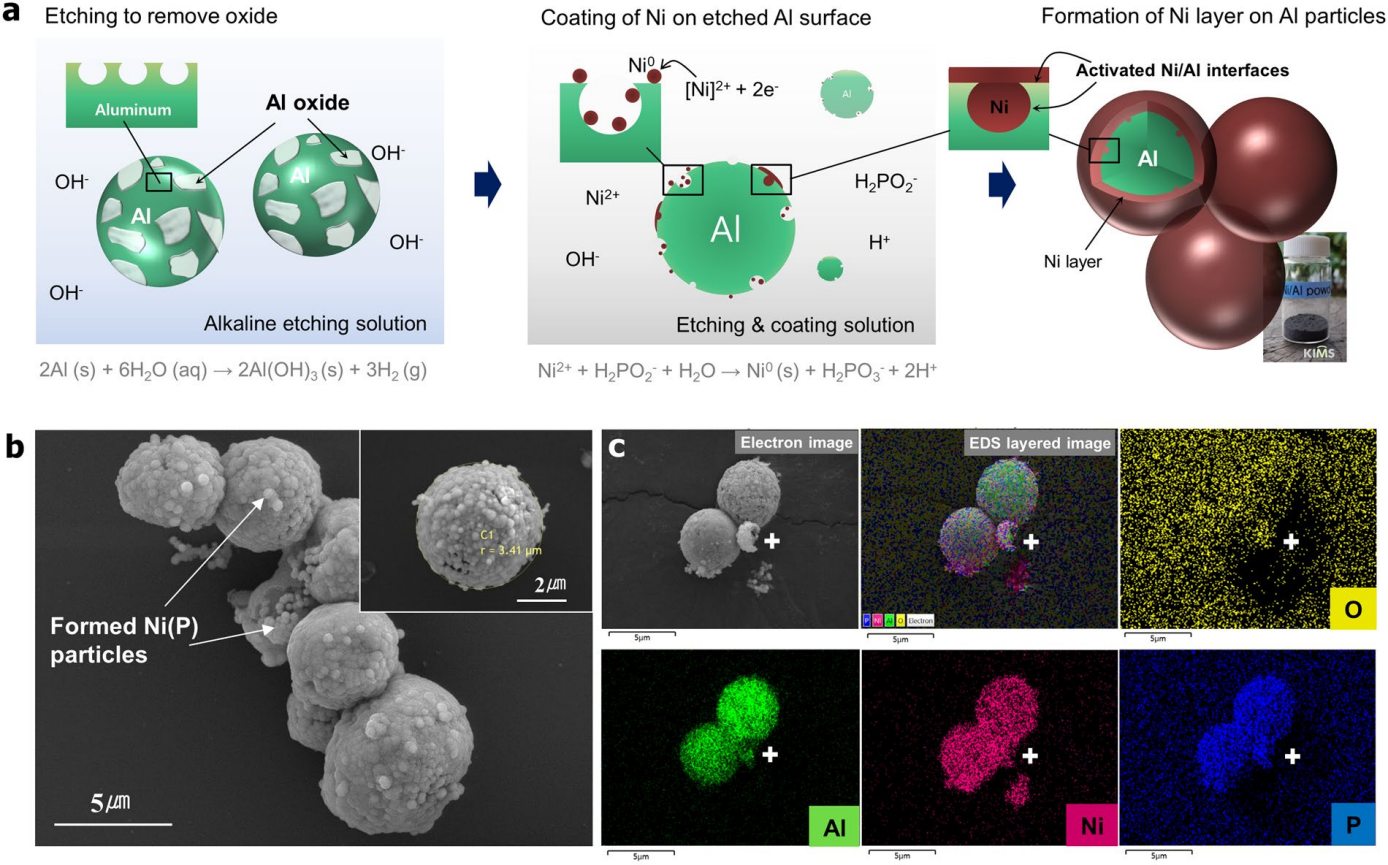
(**a**) Schematic illustration of removal of surface oxide by NaOH etching solution, forming a Ni layer on Al particles with nanosized craters dispersed on their surfaces. (**b**) Surface morphology of the synthesized Ni/Al particles. (**c**) EDS maps showing O, Al, Ni, and P distributions.

Figure 1a shows the overall synthesis process for removing the surface oxide and coating Ni onto the Al particles by electroless plating (see the experimental section for details). The surface oxides can be dissolved by using an alkaline solution, as follows:

1 $${{\rm{Al}}}_{2}{{\rm{O}}}_{3}({\rm{s}})+6{\rm{NaOH}}({\rm{aq}})\to 2{\rm{Al}}{({\rm{OH}})}_{3}({\rm{s}})+3{{\rm{Na}}}_{2}{\rm{O}}\,({\rm{s}})$$

Then, hydrogen gas is vigorously generated at the Al_2_O_3_/Al interface according to^28^

2 $$2{\rm{Al}}({\rm{s}})+6{{\rm{H}}}_{2}{\rm{O}}({\rm{aq}})\to 2{\rm{Al}}{({\rm{OH}})}_{3}({\rm{s}})+3{{\rm{H}}}_{2}({\rm{g}})\uparrow $$

The gas formed at the interface is diffused outward as the surface layer of the alumina is removed by the pressure generated by gas expansion. Simultaneously, the newly formed nanosized holes increase the specific surface area because the surface oxides are peeled off forcefully rather than being smoothly removed.

Subsequently, the ionized Ni^2+^ cations are reduced to Ni^0^ on the Al surfaces and enter the pits created by the etching process, as shown schematically in Fig. 1a. Figure 1b shows the surface morphology of the synthesized Ni/Al particles. A particle size of approximately 5 μm is obtained despite etching process. The surface of the coated Al powder is rough owing to the presence of adsorbed Ni particles nucleated by the coating process. Figure 1c shows the energy-dispersive X-ray spectroscopy (EDS) mapping results, which reveal that Ni, Al, and P atoms are homogeneously distributed on the Ni/Al particles, whereas the distribution of O atoms is inhomogeneous. Hypophosphate acts as a reducing agent for Ni ions and is nucleated with Ni nanoparticles during the coating process. Thus, P atoms remain in the powder.

**Figure 2:**
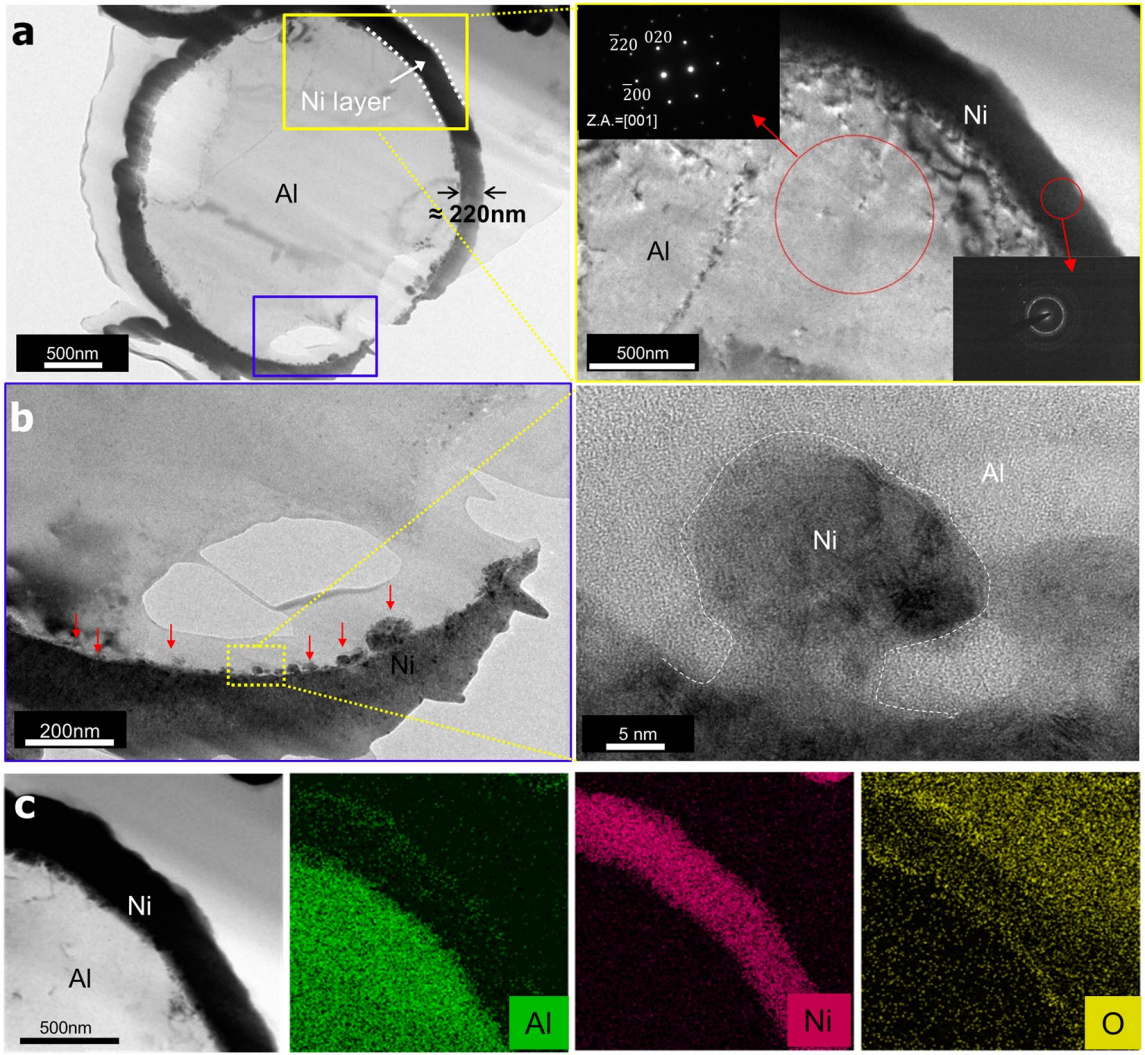
(**a**) Cross-sectional TEM image of Ni/Al particle and enlarged microstructure of zone indicated by the yellow box. (**b**) Enlarged TEM image of the zone indicated by the blue box in (**a**) to show the rough Ni/Al interface and nanosized crater filled by Ni material. (**c**) EDS maps of Ni/Al particles showing the distributions of Al, O, and Ni atoms.

Figure 2 shows bright-field transmission electron microscopy (BF-TEM) images of the Ni/Al particles prepared by the focused ion beam (FIB) technique. The Ni layer, which appears as a dark contrast, is uniformly coated on the Al particles. Given that the measured thickness of the layer is 220 nm, the weight and atomic ratios of Al and Ni in the Ni/Al particles are calculated as 1:0.461 and 1:0.212, respectively. Figure 2b shows perfect filling of the nanosized craters on the Al surface with Ni material, and the coated Ni is detected as an amorphous phase in the high-resolution TEM images. In particular, some 2–3-nm-thick Al–O regions tend to be somewhat observed near the surface of etched Al (Fig. S1 in the Supplementary Information). Considering that Al_2_O_3_ films are often more than 4 nm in thickness, this abnormally thin oxide layer is thought to originate from the repassivation of Al during the synthesis. Figure 2c shows that there are slightly more oxygen atoms around the Ni/Al interface than in the Ni layer far from the Al core. This behavior might be attributable to the remnant aluminum hydroxide formed during etching or repassivation of the Al surface.

**Figure 3:**
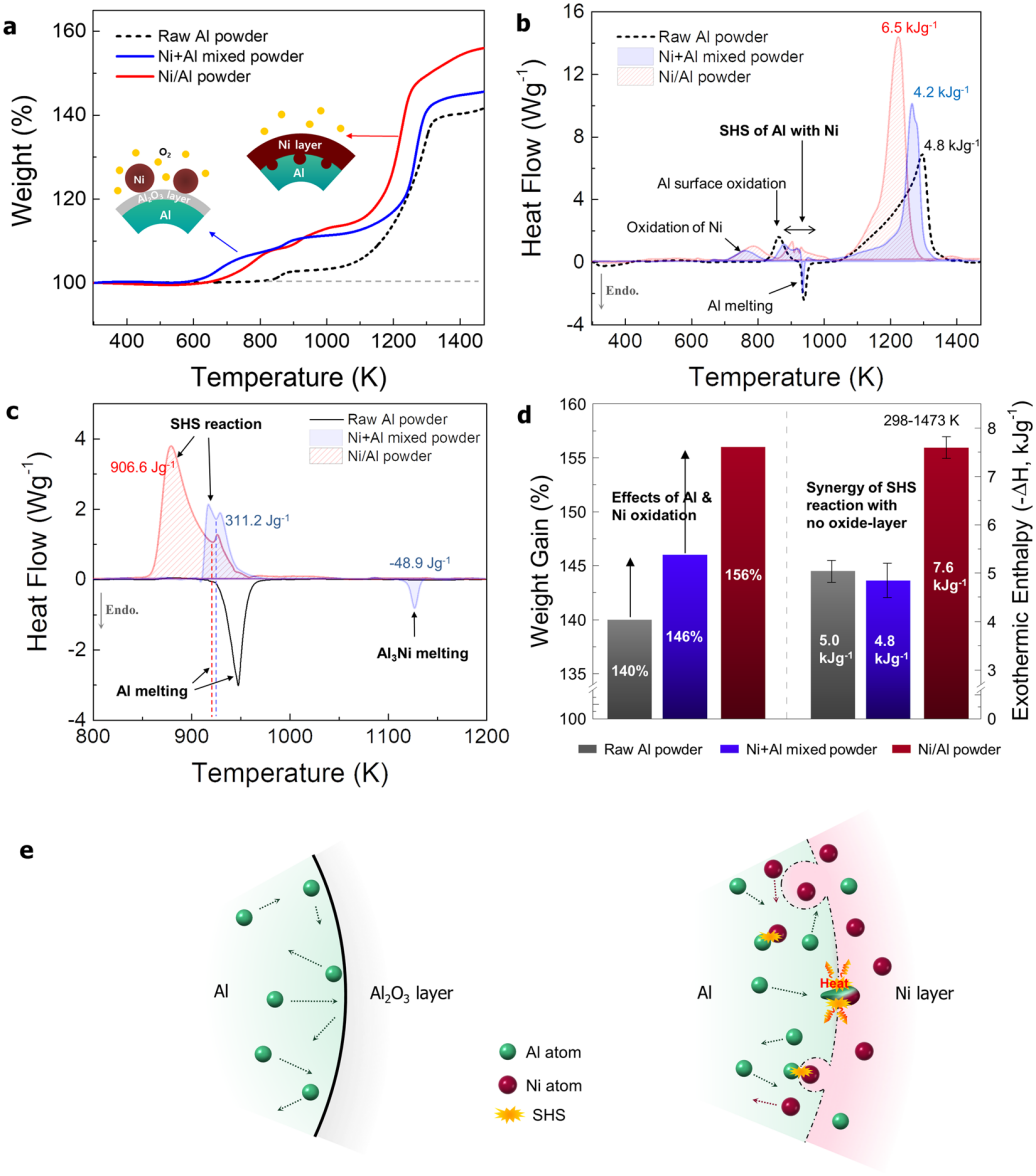
(**a**) TGA and (**b**) DSC results with increasing temperature in air atmosphere (heating rate of 10 K min^−1^), (**c**) changes in heat flow as a function of temperature in Ar atmosphere (heating rate of 10 K min^−1^), (**d**) comparison of weight gain and exothermic enthalpy energy of fine Al particles, mixed powder, and Ni/Al powder, (**e**) schematic illustration of SHS reaction in activated Ni/Al interface compared to that in surface-passivated Al.

Figure 3a,b show the results of thermogravimetric analysis (TGA) and differential scanning calorimetry (DSC), respectively, performed on the Ni/Al powder as a function of temperature from 293 to 1473 K. In addition, a sample was prepared by mixing 200-nm Ni powder and alumina-passivated fine Al powder, and its TGA and DSC results were obtained to confirm the effect of the Ni coating. The weight ratio of Ni and Al in the mixed powder was set to 1:0.46 so that the preparation conditions would be similar to those of the Ni/Al powder. As shown in Fig. 3a, the weight gain of the mixed powder starts at 600 K; i.e., it is slightly faster than that of the Ni/Al powder (where the weight gain begins at approximately 650 K) because of the easier oxidation of Ni nanoparticles in the mixed powder. Because of thermal oxidation, the Ni/Al powder shows the highest weight gain of 156% near 1473 K compared to those of the mixed powder and alumina-passivated fine Al powder. As shown in Fig. 3b, the Ni/Al powder shows the largest exothermic reaction peak at 1000–1350 K; additional exothermic peaks that appear near 850–1000 K may be related to the formation of intermetallic compounds.

The Ni/Al powder shows a large-area exothermic peak at 850–950 K in an inert gas atmosphere, in contrast to the small peak in a narrow temperature range of 900–960 K in the mixed powder, as shown in Fig. 3c. The peak areas for the Ni/Al and mixed powders are converted into exothermic enthalpy energies of 906 and 311 J g^−1^, respectively. That is, despite the inert gas atmosphere, both the Ni/Al and mixed powders show exothermic reactions below 1000 K due to the SHS reaction. In particular, the DSC peak at 1120 K in Fig. 3c reveals the formation of the NiAl_3_ phase in the mixed powder. Figure 3d compares the quantitative weight gain and exothermic enthalpy energy obtained from the TGA and DSC profiles of the fine Al powder, mixed powder, and Ni/Al powder. When the Ni nanoparticles are added, the weight gain of the mixed powder becomes 146%, which exceeds that of the Al powder (140%). Furthermore, the Ni-coated fine Al powder exhibits the highest weight gain of 156%. That is, the Ni coating enhances the efficiency of thermal oxidation of the Al material. The exothermic enthalpy energies of the Al and mixed powders are similar, approximately 5 kJ g^−1^. In addition, the Ni/Al powder shows a significantly higher exothermic enthalpy energy of 7.6 kJ g^−1^, which is 1.5 times higher than that of the Al powder. These results imply that the Ni coating on the surface-oxide-minimized Al powder allows for effective thermal oxidation of the Al material per unit weight. As shown schematically in Fig. 3e, the SHS of the mixed powder has a slower start and lower exothermic heat than that of the Ni/Al powder, presumably because of the difference between the ease of diffusion of Al and Ni atoms^8^. That is, whereas the diffusion of Al atoms is limited by the presence of Al_2_O_3_ in the simple mixed powder, direct contact between Ni and Al in the coated powder is expected to accelerate an active and efficient reaction to produce intermetallic compounds, resulting in rapid exothermic energy production. Further exothermic reaction depends on the heat energy resulting from the formation of NiAl_3_, Ni_2_Al_3_, and NiAl^29^.

**Figure 4:**
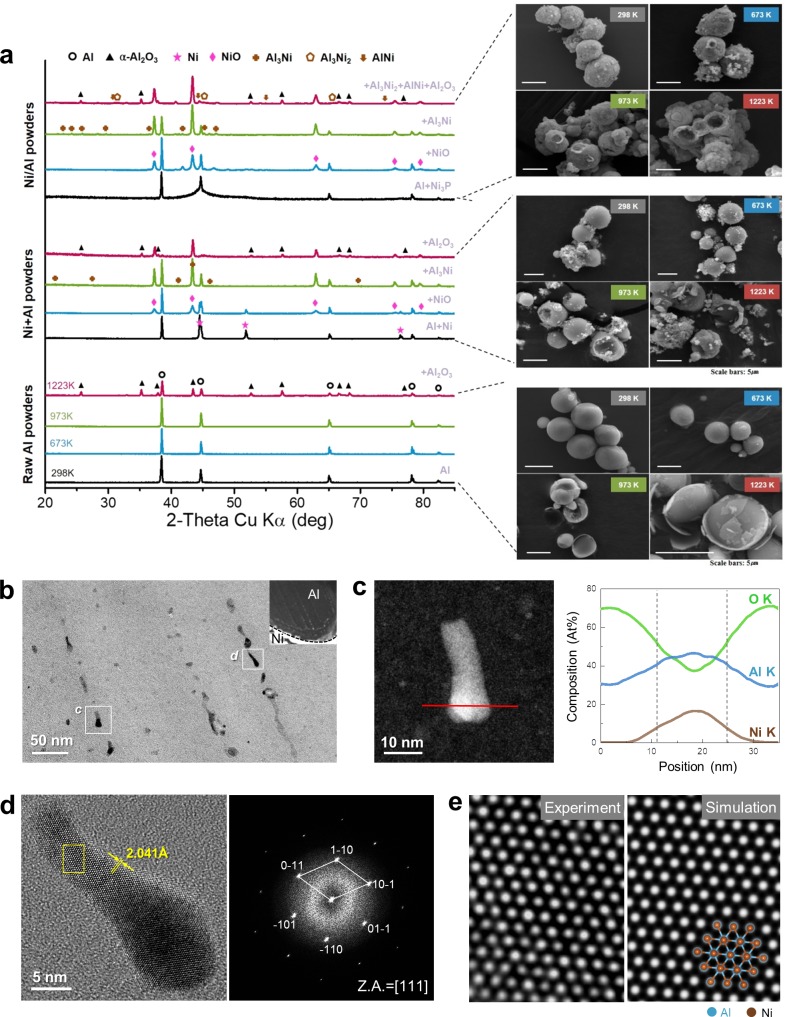
(**a**) XRD patterns of Ni/Al powder, Ni + Al mixed powder, and Al powder; surface morphologies of Ni/Al particles, Ni + Al mixed powder, and Al powder characterized after thermal oxidation at 298, 673, 973, and 1223 K in air atmosphere. (**b**) Conventional BF-TEM image and STEM-HAADF image (inset) of Ni/Al particle under heat treatment during TEM observation. (**c**) STEM-HAADF image of individual intermetallic compound and EDS line profiles of O K (yellow), Al K (green), and Ni K (brown) taken along the red line. (**d**) Experimental HR-TEM image of intermetallic compound along [111] viewing direction and fast-Fourier-transformed diffraction pattern obtained from HR-TEM image. (**e**) Enlarged HR-TEM image of the region marked in (**d**) and simulated image (defocus 10 nm, *t* = 6 nm). A schematic of the crystal structure is superimposed.

Figure 4a compares the X-ray diffraction (XRD) patterns and surface morphologies of all the samples after thermal oxidation at 298, 673, 973, and 1223 K (see Figs S2–S4 for details). The XRD patterns of the Al powder at 298, 673, and 973 K show no phase differences, but the profiles of the Ni/Al and mixed powders show the formation of NiO phases starting at 673 K. This result clarifies that the initial weight gain near 650 K in Fig. 3a is due to the oxidation of Ni in the mixed and Ni/Al powders. The formation of an intermetallic compound, NiAl_3_, is also revealed in the XRD profiles of both powders after oxidation at 973 K. All the samples show the formation of a crystalline α-Al_2_O_3_ phase after thermal oxidation at 1223 K. The surface morphologies of the powders upon thermal oxidation at each temperature indicate that the Al is oxidized to a greater extent than the alumina-passivated Al powder when Ni is added by coating or mixing. Therefore, hollow particles that might be formed under severe oxidation conditions such as explosions are not observed in the Al powder. However, other intermetallic compounds such as NiAl and Ni_2_Al_3_ are detected only in the oxidation results of the Ni/Al powder and not in those of the mixed powder. This can be attributed to the limited atomic diffusion through the Al_2_O_3_ film in the case of the mixed powder, even though Al existed in the molten phase. The thermodynamic formation and exothermic enthalpy energies of the intermetallic compounds can be summarized as follows^21^:

3 $${\rm{Al}}({\rm{s}})+{\rm{Ni}}({\rm{s}})\to {{\rm{Al}}}_{3}{\rm{Ni}}+150.6\,{\rm{kJ}}\,{{\rm{mol}}}^{-1}$$

4 $${\rm{Al}}({\rm{l}})+{\rm{Ni}}({\rm{s}})\to {{\rm{Al}}}_{3}{{\rm{Ni}}}_{2}+293.2\,{\rm{kJ}}\,{{\rm{mol}}}^{-1}$$

5 $${\rm{Ni}}+{{\rm{Ni}}}_{2}{{\rm{Al}}}_{3}\to {\rm{AlNi}}+129.2\,{\rm{kJ}}\,{{\rm{mol}}}^{-1}$$

According to these reactions, the NiAl_3_ phase forms first, and molten Al easily reacts with the Ni layer to form Ni_2_Al_3_; subsequently, NiAl is formed as a thermodynamically stable phase with increasing temperature.

Figure 4b shows a scanning transmission electron microscopy (STEM) high-angle annular dark-field (HAADF) image (inset) and enlarged BF-TEM image of a heat-treated Ni/Al particle that was slowly heated to 873 K and cooled to 300 K during the TEM observation (see the experimental section for more details). Owing to this heat treatment, intermetallic precipitates with irregular shapes and sizes of 10–20 nm were formed within the Al matrix. The chemical composition of these intermetallic precipitates was identified using EDS line analysis in STEM, which showed that Ni and Al are present in an atomic ratio of approximately 1:1 (AlNi) (Fig. 4c). A high-resolution (HR) TEM image and the corresponding fast-Fourier-transformed diffraction pattern clearly confirmed that the observed intermetallic compound is indeed AlNi. The measured *d*-spacing is ~2.041 Å, which corresponds to the {110} plane of cubic AlNi (Fig. 4d). A simulated HR-TEM image based on the B2 crystal structure of AlNi (*a* = 2.886 Å) is perfectly consistent with the measured HR-TEM image, as shown in Fig. 4e.

In contrast to the XRD results, the microscopy observations indicate that the AlNi intermetallic compound was formed at a relatively low temperature, owing to not only a thin-foil effect and surface diffusion but also to the high-vacuum environment (10^−5^ Pa).

Note that the formation of intermetallic compounds occurs preferentially until the ambient oxygen atoms reach the Ni/Al interface. Once oxygen comes into contact with the pure Al surface, active oxidation occurs in the Ni/Al powder. Thus, both the initial oxidation of Ni and SHS of Ni/Al accelerate the exothermic reaction for ignition, triggering further oxidation of Al by inducing active diffusion of Al atoms during oxidation.

## Conclusoins

In summary, densely Ni-layer-coated Al powder with an average particle size of 5 μm is synthesized by a proposed electroless plating technique. The Ni coating layer comes into direct contact with the Al surface, and the Ni/Al interface is rough because of the inhomogeneous removal of the surface oxide. Because of the direct Ni/Al interface, the exothermic energy released from the Ni/Al powder is 1.5 times higher than that of the uncoated Al powder and a mixed powder below 1473 K. Thermal analysis clearly shows the enhanced exothermic reactivity of the Ni/Al powder. It is confirmed that there is a suitable balance between the rapid oxidation of Al and further energy release by the formation of intermetallic compounds in the Ni-coated Al powder. These results can be used not only to develop advanced energetic materials with enhanced reactivity performance but also to elucidate the oxidation mechanism, which exploits the effect of the SHS reaction in reactive metallic powders. In addition, these findings on the novel Al particles with an activated Ni/Al interface can be directly applied not only in the cutting-edge design of energetic materials such as metal fuels or propellants, but also in high-performance structural materials that require aluminide intermetallic compounds.

## Methods

### Chemicals and materials

Al powder having spherical particles with an average size of 5 µm was used. The surface oxide was approximately 8.6 nm thick (Fig. S5 in the Supplementary Information). Nickel(II) sulfate hexahydrate (NiSO_4_·6H_2_O), sodium hypophosphite monohydrate (NaH_2_PO_2_·H_2_O), and sodium citrate dihydrate (Na_3_C_6_H_5_O_7_·2H_2_O) as the complexing agent and maleic acid (C_4_H_4_O_4_) as the stabilizer were purchased from Sigma-Aldrich. The pH was adjusted using ammonium hydroxide solution (NH_4_OH, 30 wt%).

### Preparation of Ni-coated Al powder

An alkaline etching solution (50 mL) was prepared using 45 mL of 5.0 mmol L^−1^ sodium hydroxide solution and 5 mL of 30 wt% ammonia solution, after which 1.0 g of Al powder was dispersed in the solution. Approximately 2 min later, 100 mL of a nickel phosphorus coating solution was poured into the etched Al suspension, followed by mechanical stirring for 40 min. The temperature was controlled at 333 ± 2 K, and the pH was adjusted to 8.2–9.2 using an ammonia solution. The nickel phosphorus solution was composed of 0.1 mol L^−1^ nickel sulfate hexahydrate, 0.4 mol L^−1^ sodium hypophosphite monohydrate, 0.15 mol L^−1^ sodium citrate dihydrate, and 5.0 mg L^−1^ maleic acid in 100 mL of distilled water. The synthesized Ni–P/Al powder was filtered and washed with deionized water several times and finally vacuum-dried at 333 K for 24 h.

### Characterization

The surface morphology of the powders was characterized by field-emission scanning electron microscopy (FE-SEM, MIRA II LMH, Tescan). The local composition and elemental distribution on the particle surface were analyzed by FE-SEM with EDS. The phases of the powders were analyzed by XRD (Rigaku International Co., D/Max-2500VL). TGA and DSC (TA Instruments, Q600) were conducted at a heating rate of 10 K min^−1^ from 298 to 1473 K in air or argon atmosphere.

A cross-sectional specimen of Ni/Al powder for TEM observation was prepared using a FIB (NOVA200, FEI Inc.) lift-off technique. A field-emission transmission electron microscope operated at 200 kV (JEM-2100F, JEOL) was used for the microstructural analysis of the Ni/Al interface. To identify the chemical compositions of the intermetallic compounds, STEM-HAADF imaging and elemental mapping using EDS (X-Max 100, Oxford instruments) were performed using a Cs-corrected scanning transmission electron microscope (JEOL 2100 F). For the *in-situ* heating TEM experiments, a cross-sectional TEM sample of Ni/Al particle was heated to 873 K at 5°/min using a Gatan 652-Ta double-tilt heating holder and cooled to room temperature. The enthalpies of the powders subjected to thermal analysis were calculated from the peak area under the DSC curves. The DSC equipment was calibrated for temperature and enthalpy using indium as a standard material according to the ASTM E967 and E968 guidelines.

## Electronic supplementary material

Supplementary information
