# Supplementary material for: Self-Propagating Heat Synthetic Reactivity of Fine Aluminum Particles via Spontaneously Coated Nickel Layer
Source: Sci Rep. 2019 Jan 31;9:1033. doi: 10.1038/s41598-018-36760-y (PMC6355937; doi:10.1038/s41598-018-36760-y)
Supplement: Supplementary file 1 — Supplementary information [file 41598_2018_36760_MOESM1_ESM.doc]

Supplementary Information

Self-Propagating Heat Synthetic Reactivity of Fine Aluminum Particles via Spontaneously Coated Nickel Layer

Dong Won Kim,^†^ Kyung Tae Kim,^*,†^ Gu Hyun Kwon,^†^ Kyung Song,^†^and Injoon Son^*,‡^

^†^ Korea Institue of Materials Science, 797 Changwondaero, Seongsan-gu, Changwon, Gyeongnam 51508, Republic of Korea

^‡^ Kyungpook National University, 80 Daehakro, Buk-gu, Daegu 41566, Republic of Korea

^*^Corresponding Authors: E-mail: ktkim@kims.re.kr; E-mail: ijson@knu.ac.kr


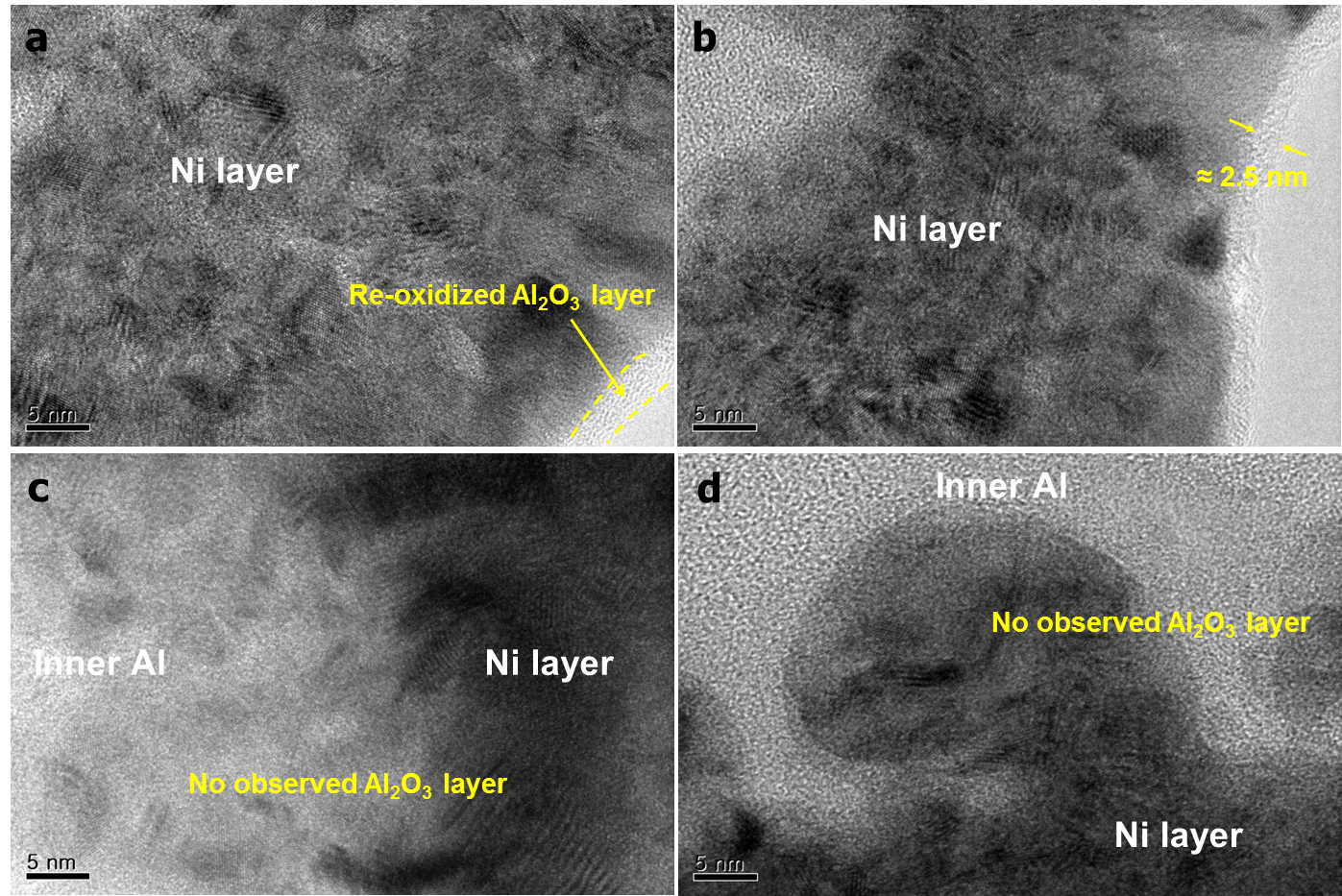


Figure S1. Cross-sectional TEM images of multiple areas such as (a, b) re-oxidation part, (c) no-oxide layer part and (d) Al surface pore part at Ni/Al interfaces.


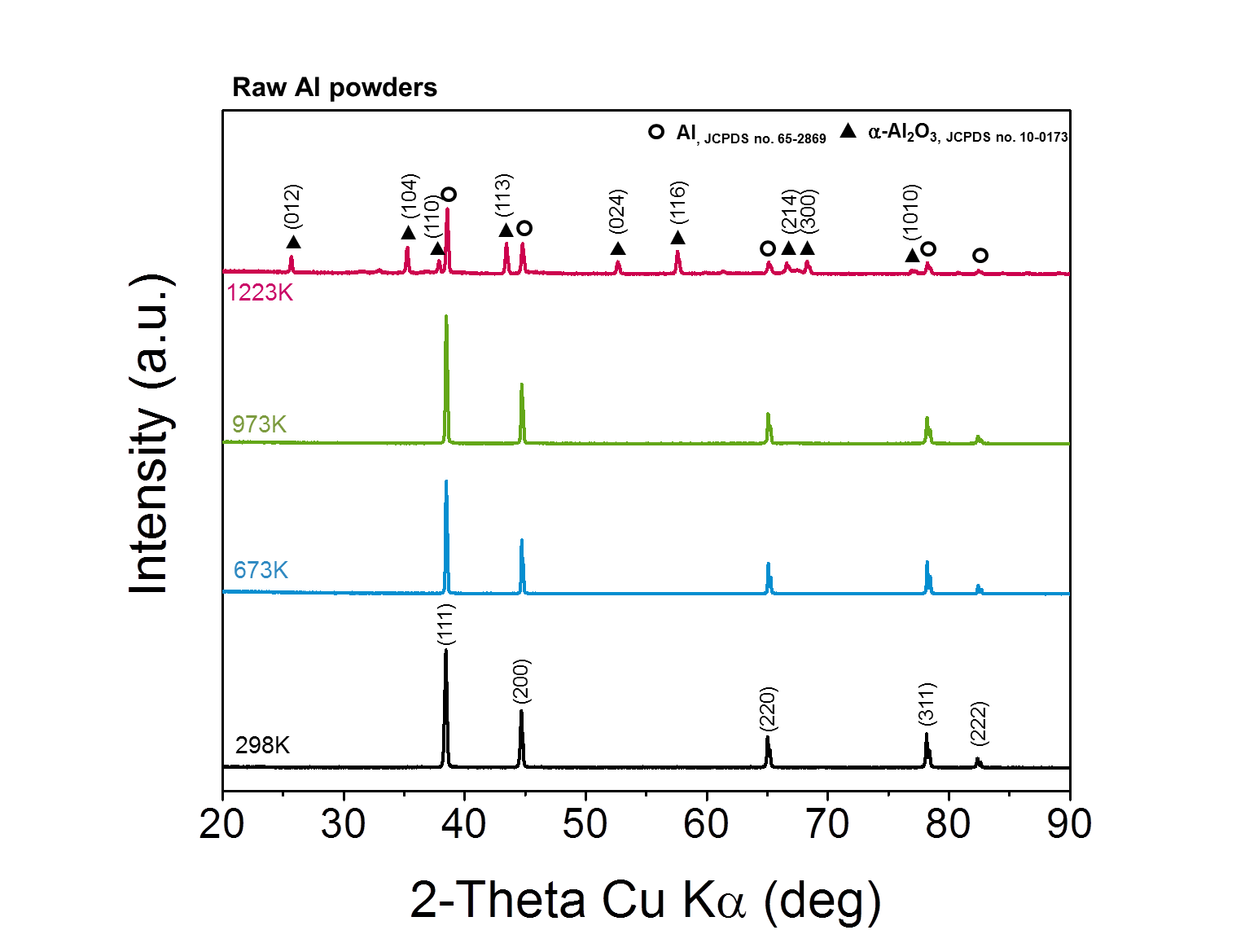


Figure S2. X-ray diffraction patterns of the raw Al powders heat-treated at 673 K, 973 K and 1223 K for 30 min under air atmosphere.


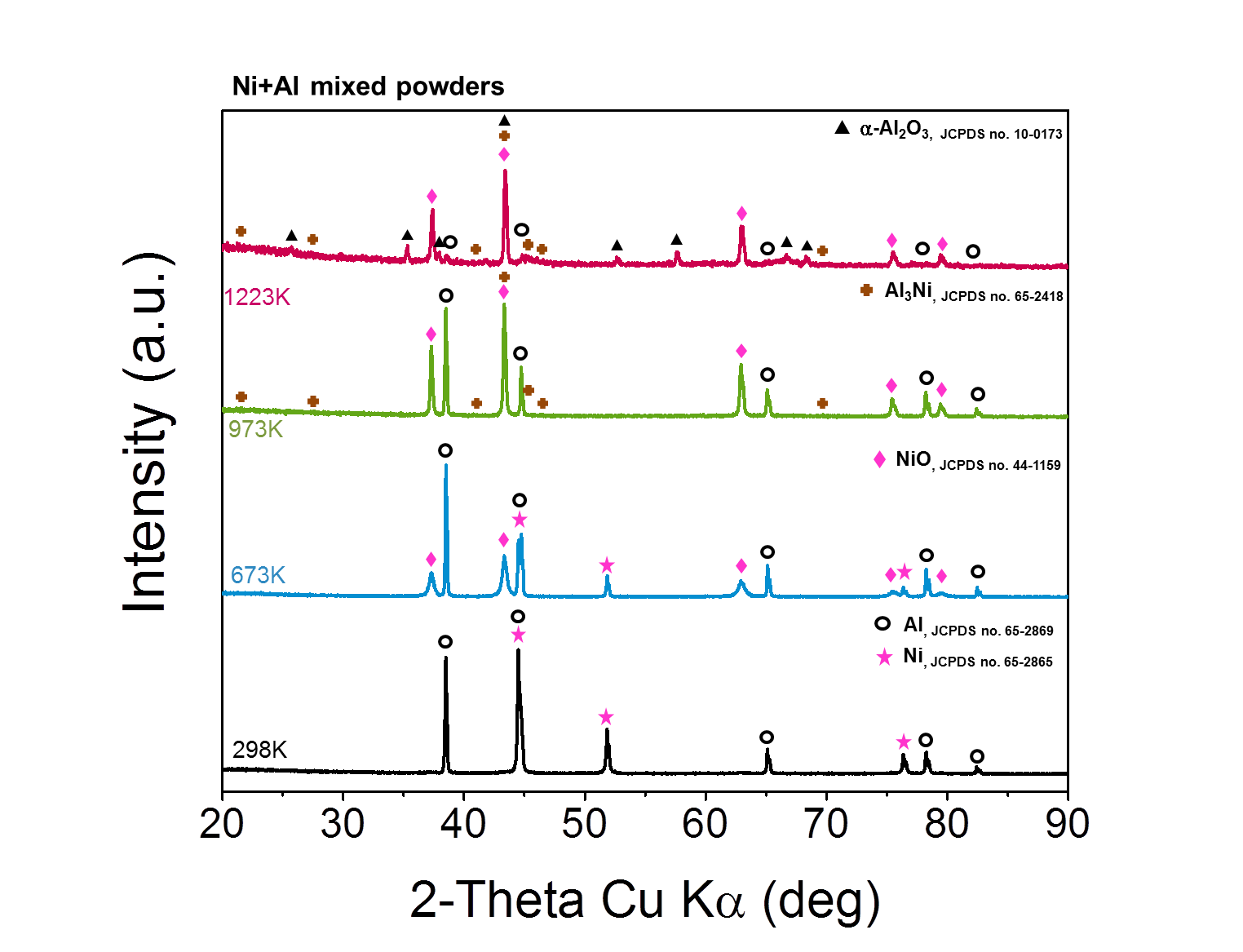


Figure S3. X-ray diffraction patterns of the mixed powders heat-treated at 673 K, 973 K and 1223 K for 30 min under air atmosphere.


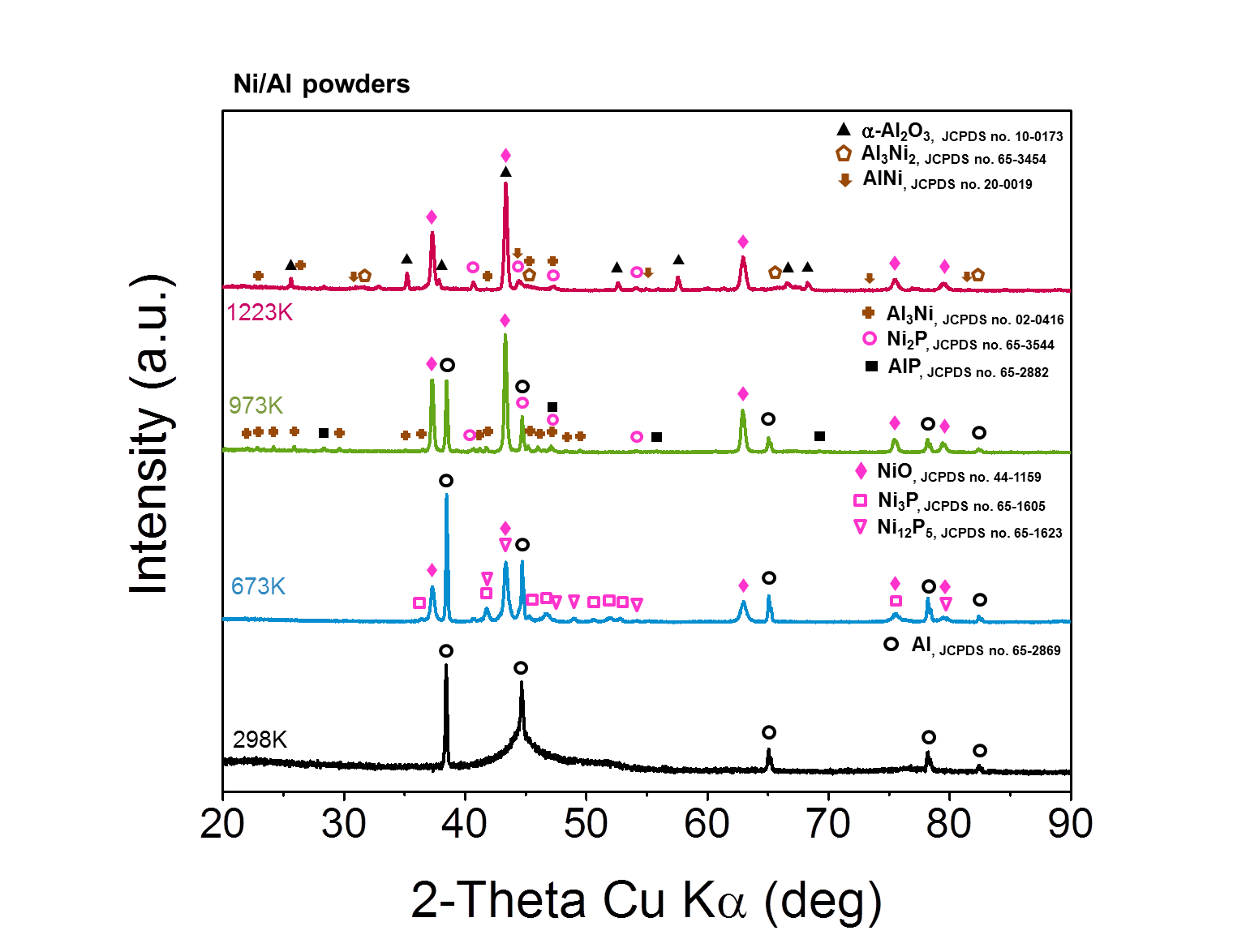


Figure S4. X-ray diffraction patterns of the Ni/Al powders heat-treated at 673 K, 973 K and 1223 K for 30 min under air atmosphere.

The surface atoms of amorphous Ni-P are oxidized at 673 K to form NiO and become Ni_12_P_5_ phase. And Ni_3_P appears to be crystallized. Intermetallic Al_3_Ni compound is formed by the SHS reaction of nickel and aluminum at 973 K, which in this SHS reaction is converted to Ni_2_P phase due to nickel consumption of Ni_3_P. Accordingly, aluminum reacts with phosphorus to form an AlP compound as shown in the following equation (S1):

4Al + P_4_ → 4AlP(s), ∆H^0^_298_ = -164.4 kJmol^-1 [1]^ (S1)


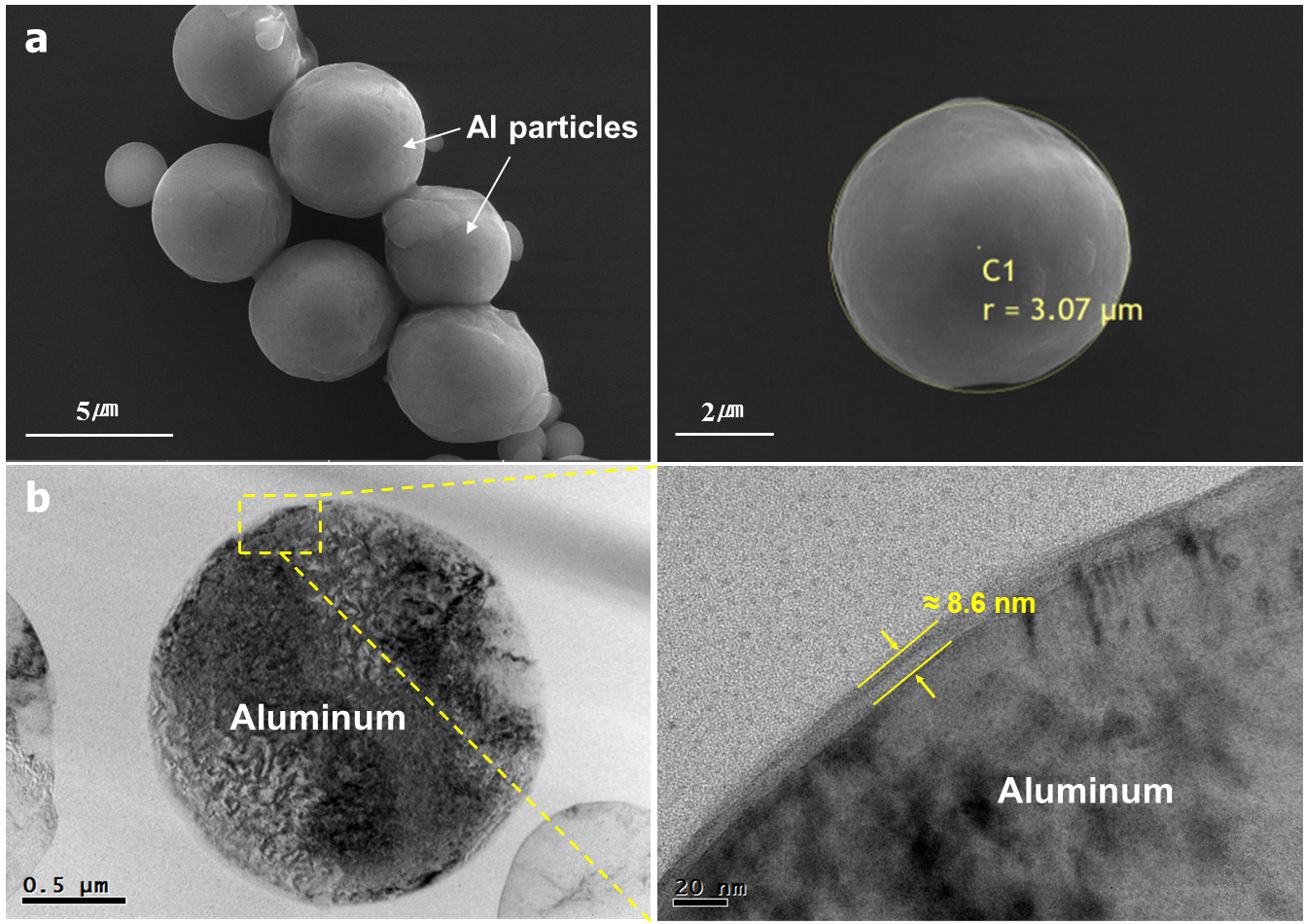


Figure S5. (a) FE-SEM and (b) cross-sectional TEM images of raw Al powder used in this work.

The Al particles exhibit a size distribution of to 2 to 7 um, and the oxide layer thickness of Al particle is about 8.6 nm as shown the cross-sectional TEM image.


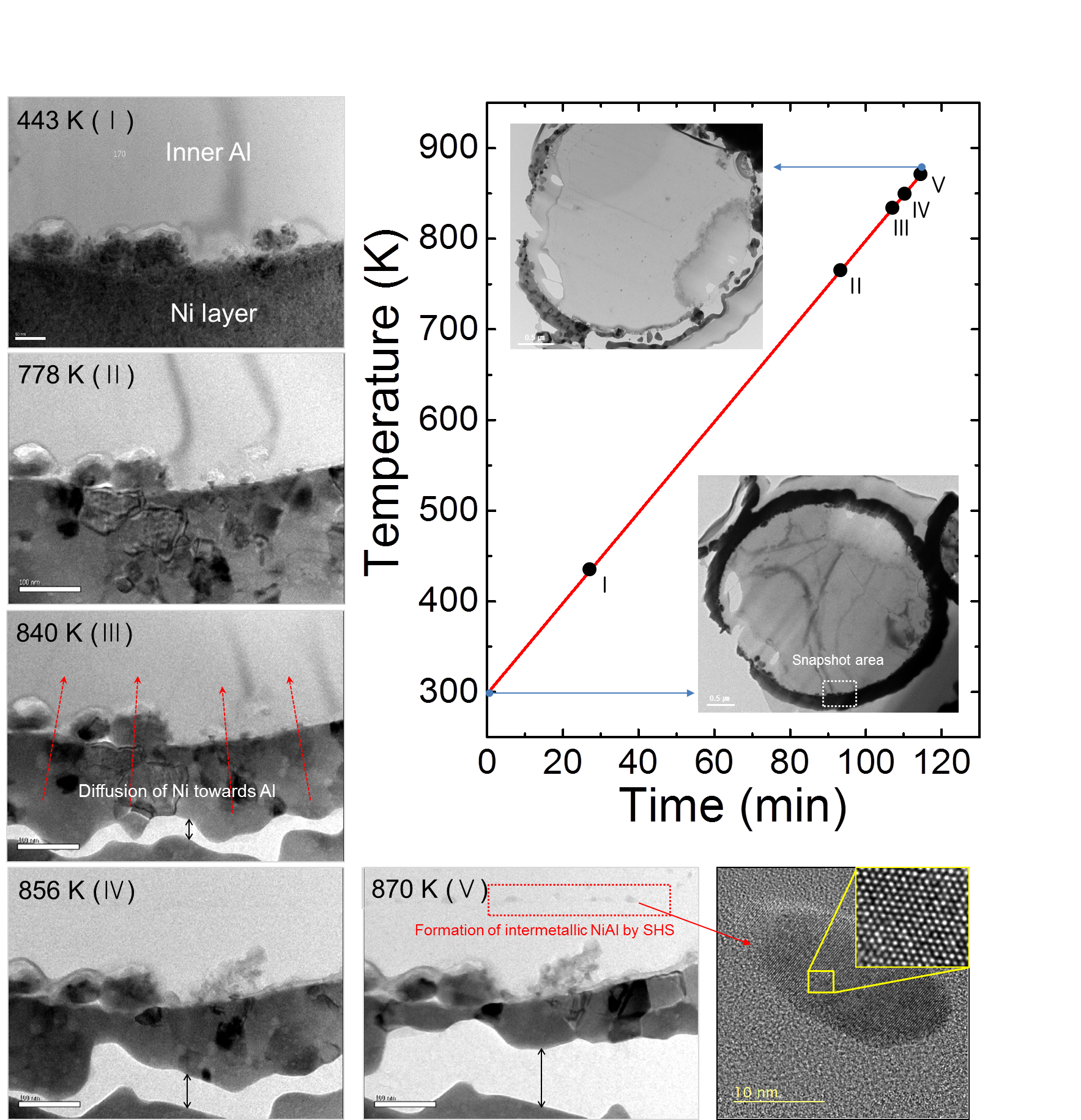


Figure S6. Snapshots of the Ni/Al interfacial area due to increasing temperature at in-situ heating TEM experiment.

At 840 K, cracks began to appear in the Ni layer, and it was observed in real time that the thickness of the Ni layer became thinner. It is assumed that as the Al melts at about 840 K, the Ni atoms diffuse toward the inner Al. Subsequently, an intermetallic compound formed by SHS in the Al internal region was identified at 870 K.

**Reference**

[1] M. Binnewies, E. Milke, *Thermochemical Data of Elements and Compounds,* Second, Revised and Extended Edition ed., Wiley-VCH, Weinheim, **2002**.
